# Lithium-Excess Research of Cathode Material Li_2_MnTiO_4_ for Lithium-Ion Batteries

**DOI:** 10.3390/nano5041985

**Published:** 2015-11-20

**Authors:** Xinyi Zhang, Le Yang, Feng Hao, Haosen Chen, Meng Yang, Daining Fang

**Affiliations:** 1 College of Engineering, Peking University, Beijing 100087, China; E-Mails: zhxy235@pku.edu.cn (X.Z.); leyang@pku.edu.cn (L.Y.); fangdn@pku.edu.cn (D.F.); 2 Department of Earth and Environmental Engineering, Columbia University, New York, NY 10027, USA; E-Mail: fh2314@columbia.edu; 3 Institute of Advanced Structure Technology, Beijing Institute of Technology, Beijing 100081, China; 4 College of Materials Science and Engineering, Nanjing Tech University, Nanjing 210009, China

**Keywords:** lithium-ion batteries, cathode, titanate material, nanoscale particle, lithium excess, phase transition

## Abstract

Lithium-excess and nano-sized Li_2+x_Mn_1__−*x*/2_TiO_4_ (*x* = 0, 0.2, 0.4) cathode materials were synthesized via a sol-gel method. The X-ray diffraction (XRD) experiments indicate that the obtained main phases of Li_2.0_MnTiO_4_ and the lithium-excess materials are monoclinic and cubic, respectively. The scanning electron microscope (SEM) images show that the as-prepared particles are well distributed and the primary particles have an average size of about 20–30 nm. The further electrochemical tests reveal that the charge-discharge performance of the material improves remarkably with the lithium content increasing. Particularly, the first discharging capacity at the current of 30 mA g^−1^ increases from 112.2 mAh g^−1^ of Li_2.0_MnTiO_4_ to 187.5 mAh g^−1^ of Li_2.4_Mn_0.8_TiO_4_. In addition, the *ex situ* XRD experiments indicate that the monoclinic Li_2_MnTiO_4_ tends to transform to an amorphous state with the extraction of lithium ions, while the cubic Li_2_MnTiO_4_ phase shows better structural reversibility and stability.

## 1. Introduction

Lithium-ion batteries (LiBs) have been widely used as an environmentally friendly energy storage and release system, and in recent years the need for LiBs with higher performance has become increasingly urgent due to the demand of electric vehicles and the storage of new energy [1]. As the cathode is a major component of LiBs and its performance has a vital role, developing better cathode materials is a foremost way to meet this need. The ideal cathode materials should satisfy several characteristics [2]: (1) allowing an insertion and extraction of a large amount of lithium to maximize the capacity; (2) having a large amount of ion channels and ion vacancy to allow the rapid migration of lithium ions; (3) having a stable structure to guarantee the stability of capacity. So far, lithium-transition metal oxides have represented the most mature cathode materials, which mainly include layered LiMO_2_ (such as LiCoO_2_, LiNiO_2_, LiMnO_2_ or their solid solution) and spinel-type LiMn_2_O_4_ [3,4,5,6]. All of these materials are well-ordered materials in which lithium ions and other cations occupy distinct sites. In 2003, for the first time, cation-disordered materials were investigated as cathode materials [7]. Li_2_MTiO_4_ (M = Mn, Fe, Co, Ni) materials as the representative cation-disordered materials have attracted much attention due to their high theoretical capacity (about 290 mAh g^−1^) and stability of structure [7].

The Li_2_MTiO_4_ material has a cubic cation-disordered rock salt structure with the Fm-3m space group, which means that all the metal atoms disorderly occupy the octahedral sites of the cubic close-packed array of anions. Particularly the involved strong Ti–O bond could stabilize the M^3+^/M^2+^and M^4+^/M^3+^ transition. The present Li_2_MTiO_4_ materials mainly include Li_2_NiTiO_4_, Li_2_FeTiO_4_, Li_2_MnTiO_4_, Li_2__−*x*_VTiO_4_, and Li_2_CoTiO_4_ [8,9,10,11,12,13,14,15]. The electrochemical properties indicate that Li_2_NiTiO_4_ shows a reversible capacity of 179 mAh g^−1^, which is 61.6% of the theoretical value for the exchange of 2 M lithium (290.6 mAh g^−1^). Li_2_FeTiO_4_ and Li_2_MnTiO_4_ show a comparable capacity of ~130 mAh g^−1^ if they are prepared within the carbon coating. In the case of Li_2__−*x*_VTiO_4_, the maximum capacity is close to 350 mAh g^−1^ in a wide potential range between 1.0 V and 4.4 V. For Li_2_CoTiO_4_, the discharge capacity is 150.8 mAh g^−1^ if the particle size is much smaller. Although the achieved discharge capacities of the above materials were not bad, they were still far from their theoretical values. Thus, how to enhance the discharge capacity of these materials has become an urgent and important problem. In 2014, Ceder *et al.* pointed out that increasing the amount of lithium is an effective way to improve the electrochemical properties of cation-disordered materials, which had been achieved in a Li_1.211_Mo_0.467_Cr_0.3_O_2_ material [16,17]. Inspired by this, the effect of excess lithium added to Li_2_MTiO_4_ materials is quite worthy of study.

Herein, the lithium-excess Li_2+*x*_Mn_1__−*x*/2_TiO_4_ (*x* = 0, 0.2, 0.4) cathode materials were synthesized and studied in this paper. The synthesis was done via a sol-gel method to obtain the nano-sized and well-distributed particles of the materials. The effects of different lithium amounts on the structure, morphology, and electrochemical properties were then investigated.

## 2. Results and Discussion

Three representative Li_2+*x*_Mn_1__−*x*/2_TiO_4_ materials with different lithium content were prepared, namely Li_2.0_MnTiO_4_, Li_2.2_Mn_0.9_TiO_4,_ and Li_2.4_Mn_0.8_TiO_4_. For brevity, they are respectively expressed as L2.0, L2.2, and L2.4 in the following discussions. Among them, L2.0 has a normal stoichiometric proportion while L2.2 and L2.4 contain excess lithium.

**Figure 1:**
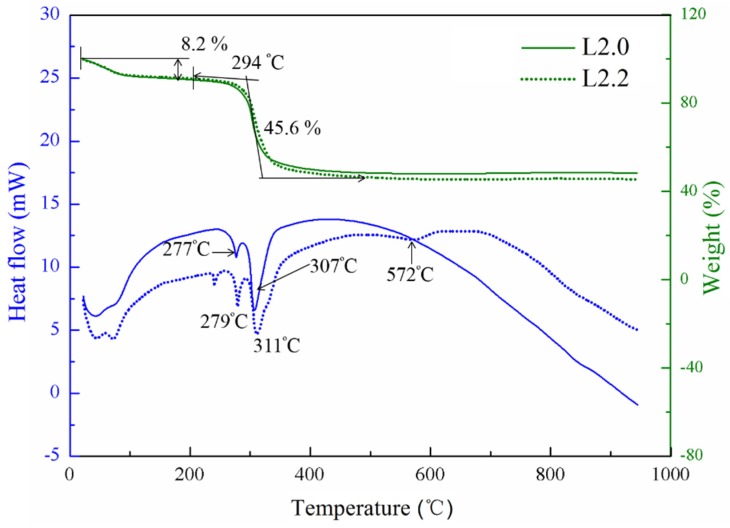
Thermogravimetry and differential scanning calorimetry (TG-DSC) curves of the L2.0 and L2.2 precursors.

The heat treatment process of the materials with different element proportions was investigated through the thermogravimetry and differential scanning calorimetry (TG-DSC) experiments. The typical curves of the precursors with normal proportion (*x* = 0, L2.0) and Li-excess proportions (*x* = 2, L2.2) are presented in Figure 1. It is found that these two samples show almost unanimous TG curves, and the mass loss of the samples can be divided into two distinct stages. At the first stage between 20 and 150 °C, the little weight loss of 8.2 wt % may be attributed to the absorbed water in the precursor, corresponding to the broad endothermic dehydration that can be found in the DSC curves. In the second stage, the two endothermic peaks occurred at about 278 °C and 310 °C and were accompanied by obvious weight loss in the TG curves. This is due to the decomposition of the inorganic and the organic component of the precursor followed by crystallization of the compound. In the region between 500 °C and 950 °C, the TG curves became flat and no weight loss could be observed. However, a weak discrepancy between these two samples can be observed from the DSC curves in this region. In the case of L2.0, the DSC curve shows a continuously endothermic process from the temperature of 500 °C to 950 °C. For L2.2, a shallow endothermic peak can be observed at 572 °C, indicating that the phase or the structure has been changed. The difference between the curves suggested that excess lithium had caused a significant impact to this kind of material.

**Figure 2:**
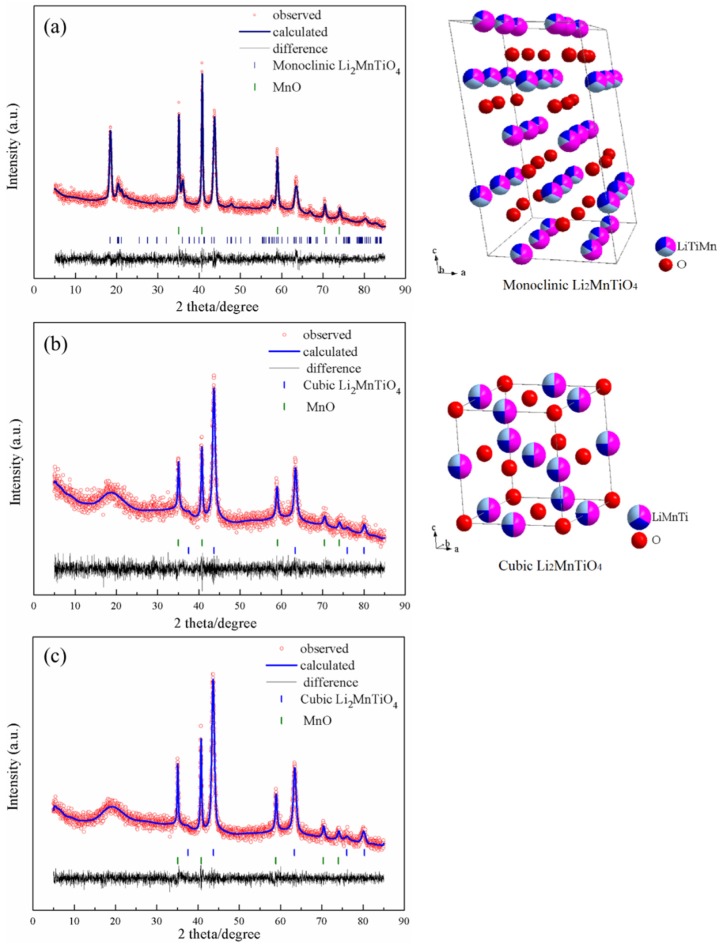
X-ray diffraction (XRD) patterns of L2.0 (**a**), L2.2 (**b**), L2.4 (**c**) and the microstructure of the involved phases. The spheres of red, pink, dark blue, and watchet blue denote the atoms of O, Li, Mn, and Ti, respectively, and the proportions of the colors correspond to the occupations of the elements.

Figure 2 shows the X-ray diffraction (XRD) patterns of as prepared Li_2+*x*_Mn_1__−_*_x_*_/2_TiO_4_ materials. Comparing the diffraction peaks of L2.0 and the Li-excess materials, a huge discrepancy in peak position can be obviously observed, indicating that different phases were obtained. Rietveld refinement of the XRD patterns shows that the L2.0 sample consisted of a monoclinic Li_2_MnTiO_4_ coexisting with MnO, while the Li-excess samples consisted of a cubic Li_2_MnTiO_4_ coexisting with MnO. In the case of L2.0, the monoclinic Li_2_MnTiO_4_ was consistent with the C2/c space group, which assumes that (Li_1/3_(Mn,Ti)_2/3_)) and (Li_2/3_(Mn,Ti)_1/3_)) cation layers alternate in the (1 1 1) cubic planes. This layer structure has a strong resemblance to monoclinic Li_2_TiO_3_ and possesses an ordered structure with lattice parameters of *a* = 5.048 Å, *b* = 8.833 Å, *c* = 9.629 Å; α = 90°, β = 99.939°, γ = 90°. A similar ordered phase of another member of the titanate family, Li_2_NiTiO_4_, was reported in a previous study [7]. For the Li-excess materials, the cubic Li_2_MnTiO_4_ was consistent with the Fm-3m space group, as previously reported [7,9]. The refined lattice parameters of L2.2 and L2.4 are, respectively, *a* = *b* = *c* = 4.151 Å; α = β = γ = 90°, and *a* = *b* = *c* = 4.151 Å; α = β = γ = 90°. Additionally, the phase contents of cubic Li_2_MnTiO_4_ of the two materials are also much the same, both about 80%. The discrepancy in the structure between L2.0 and the lithium-excess materials indicates that the cation-disordered structure can be obtained more easily through increasing the content of the lithium, which can explain the difference of the TG-DSC curves between the materials.

**Figure 3:**
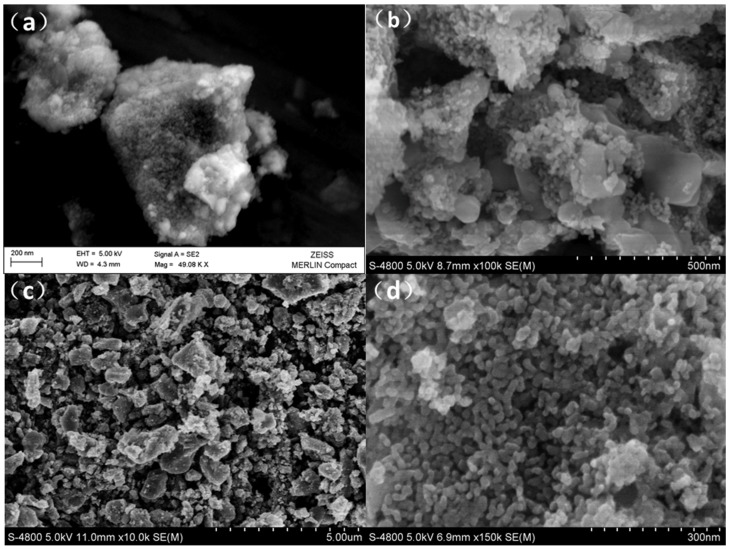
Typical scanning electron microscope (SEM) images of the as-prepared materials. (**a**) L2.0; (**b**) L2.2; (**c,d**) L2.4.

The morphology of the Li_2+*x*_Mn_1__−*x*/2_TiO_4_ materials was identified by field emission scanning electron microscope (FE-SEM) analysis, as shown in Figure 3. From the FE-SEM images, it can be found that all of the samples presented an aggregation feature, and they are composed of large numbers of small irregular particles. Most of the aggregated particles were about several hundred nanometers while the average size of the irregular primary particles was about 20–30 nm. The primary particles constructed a porous configuration which would enable us to shorten the diffusion distance of lithium ion migration and thus achieve good electrochemical performance.

**Figure 4:**
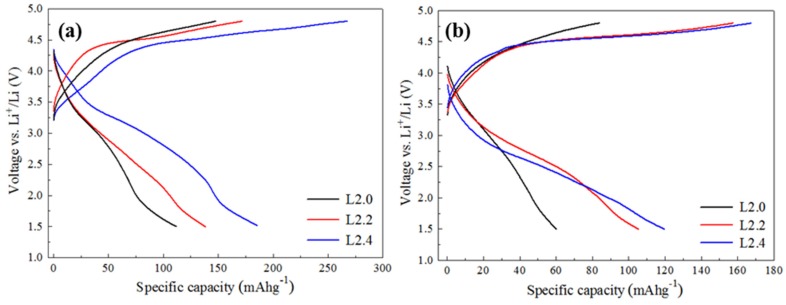
The first charge-discharge curves of the Li_2+*x*_Mn_1__−*x*/2_TiO_4_ cathode at a constant current of 30 mAg^−1^ (**a**) and 60 mAg^−1^ (**b**).

The electrochemical properties were then investigated. It should be noted that the capacity of the materials is derived from the Mn^3+^/Mn^2+^ and Mn^4+^/Mn^3+^ transition. Thus, the theoretical capacities of the L2.0, L2.2, and L2.4 can be calculated based on their Mn contents, and they are respectively 296.7 mA g^−1^, 273.2 mA g^−1^, and 248.6 mA g^−1^.Galvanostatic charge and discharge properties of the Li_2+*x*_Mn_1__−*x*/2_TiO_4_ cathode were tested between 1.5 V and 4.8 V with constant currents of 30 mA g^−1^ and 60 mA g^−1^, as shown in Figure 4. From Figure 4a, we can see that these cathodes exhibited different charge and discharge properties and the difference can be found not only in their capacities but also in their voltage plateau. In the case of the L2.0 electrode, the polarization between the charge and discharge was very large, and the discharge capacity was only 112.2 mAh g^−1^ (equivalent to 0.76 lithium per formula unit). For Li-excess electrodes, the voltage plateau of charging and discharging became more distinct, and the discharge capacity was also higher than that of L2.0. Specifically, the L2.4 electrode could deliver a capacity as high as 187.5 mAh g^−1^. When the current density increased to 60 mA g^−1^, the capacity gap between L2.0 and the lithium-excess materials became even clearer (as shown in Figure 4b). These obvious differences in electrochemical properties can be attributed to the phase transition induced by excess lithium, according to the previous discussions. In addition, by comparing the discharge capacities of L2.2 and L2.4 electrodes, we can find that they deliver different discharge capacities in spite of their identical structure and content of cubic L_2_MnTiO_4_. This may be due to the facile Li diffusion in L2.4 materials, which was corroborated by the Ceder group [16,17] who proposed that increasing Li excess can open a percolating network in disordered lithium-transition metal oxides and thus enable facile Li diffusion in these materials.

**Figure 5:**
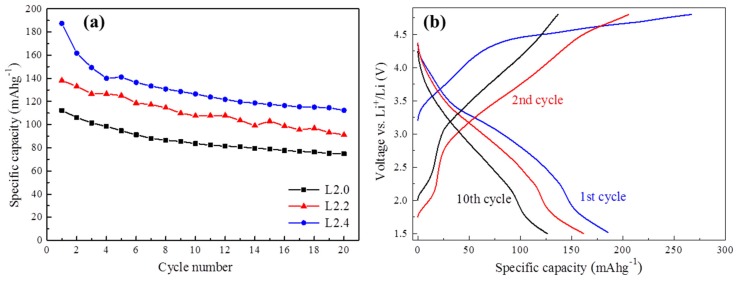
Cycling stability curves of Li_2+*x*_Mn_1__−_*_x_*_/2_TiO_4_ cathodes (**a**) and charge-discharge curves of L2.4 (**b**) at a constant current of 30 mA g^−1^.

Figure 5a shows the cycling performance of Li_2+*x*_Mn_1__−_*_x_*_/2_TiO_4_ cathodes at room temperature between 1.5 V and 4.8 V. All of the electrodes exhibited the highest discharge capacity in the first cycle. Subsequently, the capacity decreased gradually as the number of cycles increased. After 20 cycles, the capacity retentions of L2.0, L2.2, and L2.4 electrodes were respectively 66.6%, 65.9%, and 59.9%. The capacity fading of these cathode materials may be attributed to many reasons, especially the irreversible structure change during the lithium-ion deintercalation/intercalation process.

The galvanostatic load curves of L2.4 at the first, second, and the 10th cycles are exhibited in Figure 5b. It can be found that though there is an apparent capacity fading from the first to the 10th cycle, the discharge curves keep quite a similar shape, which suggests the good structural stability of the L2.4 upon cycling.

**Figure 6:**
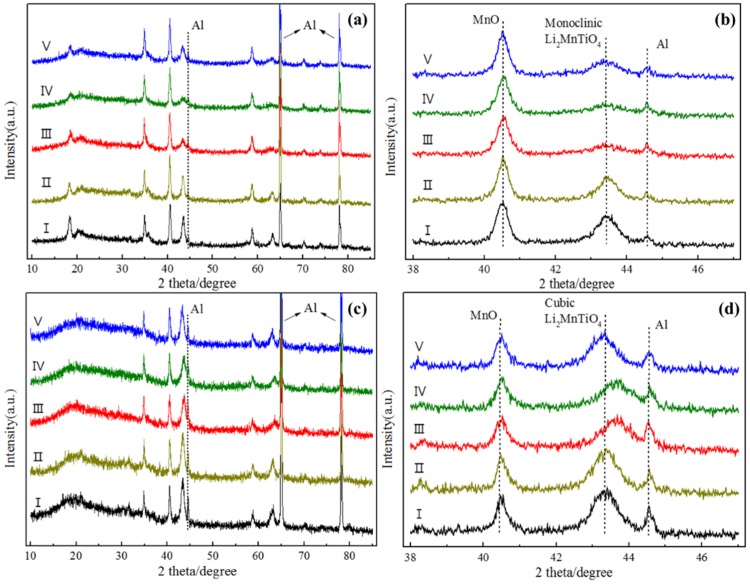
*Ex situ* XRD patterns of L2.0 (**a**,**b**) and L2.4 (**c**,**d**) electrodes during the first charge and discharge. I: initial, II: charge to 4.2 V, III: charge to 4.8 V, IV: discharge to 4.2 V, V: discharge to 1.5 V.

To investigate the microstructure changes of the electrodes during the charge and discharge process, *ex situ* XRD patterns were employed. Figure 6 shows the obtained patterns of L2.0 and L2.4 electrodes. In the case of L2.0, all of the XRD patterns (Figure 6a) at the different charge and discharge states were similar to that of the original state, and no other diffraction peaks were detected, which means that no other phases arose. However, based on the results in the enlarged image in Figure 6b, it can be found that the diffraction peak of the monoclinic Li_2_MnTiO_4_ became gentle with Li^+^ extraction during charge and did not recover completely after the subsequent discharge. This demonstrates that the crystalline monoclinic Li_2_MnTiO_4_ tends to transform to an amorphous state when the Li^+^ is extracted, suggesting that it is not stable enough to serve as a cathode material. As for L2.4, the diffraction peak of the cubic Li_2_MnTiO_4_ shifted to the higher angle compared to that of the initial state during the charge process and then moved back after the discharge. Such good structure reversibility of L2.4 indicates that the cubic Li_2_MnTiO_4_ possesses a better structural stability compared with the monoclinic Li_2_MnTiO_4_. In addition, according to the continuous change in the Bragg position, it can be inferred that the lithium exchange of the cubic Li_2_MnTiO_4_ followed the solid-solution mechanism. Based on the Retvield refinement, the change in the lattice parameters of L2.4 (volume) can be calculated. It turns out that during the first charge process, the change of the lattice constant was only 0.45%, suggesting that cubic Li_2_MnTiO_4_ could be considered as a stable cathode material for lithium-ion batteries.

## 3. Experimental

### 3.1. Synthesis

The synthesis of nanoscale Li_2+*x*_Mn_1__−*x*/2_TiO_4_ (*x* = 0, 0.2, 0.4) cathode materials was based on sol-gel reactions, followed by heat treatment under atmosphere protection. To form the sol, stoichiometric amounts of high-purity tetra-n-butyl titanate (C_16_H_36_O_4_Ti, Yonghua Chemical Technology, Suzhou, China), lithium acetate dehydrate (C_2_H_3_O_2_Li•2H_2_O, Sinopharm Chemical Reagent, Shanghai, China), and manganese acetate dehydrate (C_4_H_6_O_4_Mn•2H_2_O, Xilong Chemical, Shantou, China) were dispersed in ethanol and then continuously stirred in oil bath at 80 °C for 24 h. The sol was evaporated at 80 °C overnight and further ball-milled sufficiently to get the xerogel powders. The powders were calcined at 600 °C for 10 h in argon atmosphere to obtain the Li*_x_*Mn_2-*x*/2_TiO_4_ material.

### 3.2. Characterizations

Thermogravimetry (TG) and differential scanning calorimetry (DSC) experiments were carried out to investigate the heat treatment process of Li*_x_*Mn_2__−_*_x_*_/2_TiO_4_ materials. The precursors were heated at a rate of 10 °C min^−1^ under nitrogen atmosphere in a SDT Q600 instrument (Champaign, IL, USA) to obtain the TG-DSC curves.

The phase composition and structure of the as-prepared materials were analyzed based on powder X-ray diffraction (XRD). The diffraction data were collected on an ARL™ X'TRA Powder Diffractometer using Cu Kα radiation in the two-theta range of 10–85°. Rietveld refinement was done to achieve the analyses. To characterize the morphology, scanning electron microscopy (SEM) images of the products were collected on a HITACHI (Tokyo, Japan) S-4800 field-emission SEM (FE-SEM) at a high potential (*i.e.*, 5 kV).

Electrochemical tests were carried out using CR2032-type coin cells with Li metal as the anode. The Li_2+*x*_Mn_1__−_*_x_*_/2_TiO_4_ cathode consisted of 80 wt % of the active material, 10 wt % carbon black as conductive agent, and 10 wt % polyvinylidene fluoride (PVDF) as binder. Aluminum foils were used as the current collectors. The cells were assembled in an argon-filled glove box. The electrolyte was 1 M LiPF6 dissolved in a mixture of ethylene carbonate (EC)-Ethyl Methyl Carbonate (EMC)-dimethyl carbonate (DMC) (1:1:1, *v*/*v*). The separators were made up of Celgard3501 (USA) microporous polypropylene membrane.

The charge-discharge performance of the batteries was investigated through a BT-2000 battery testing system (Arbin, College Station, TX, USA). The tests were implemented at constant currents between voltage limits of 1.5–4.8 V at 30 °C.

## 4. Conclusions

In this paper, lithium-excess and nano-sized Li_2+*x*_Mn_1__−*x*/2_TiO_4_ (*x* = 0, 0.2, 0.4) cathode materials were successfully synthesized via a sol-gel method. The TG-DSC experiments suggest that the excess lithium induces a significant impact on the synthesis process of the material. It is confirmed by the XRD detections that the obtained main phases of L2.0 and Li-excess materials are different, namely monoclinic Li_2_MnTiO_4_ and cubic Li_2_MnTiO_4,_ respectively. The materials show similar morphology and the particles are all well distributed with an average size of about 20–30 nm. Then the electrochemical tests indicated that the charge-discharge performance of the material improves remarkably with the lithium content increasing. Particularly when the testing current density was 30 mA g^−1^, the first discharging capacity increased from 112.2 mAh g^−1^ of L2.0 to 187.5 mAh g^−1^ of L2.4. The performance improvement between L2.0 and the lithium-excess materials can be attributed to the phase transition induced by excess lithium. The improvement from L2.2 to L2.4 can be explained with the achievement of the percolating network of 0-TM channels to provide macroscopic diffusion. However, it should be noted that the Li content cannot be too high or it would lead to lower theoretical capacity and poor structural stability. Thus, there should be a limiting ratio corresponding to the optimal performance, and this is worthy of future studies. In addition, the *ex situ* XRD experiments revealed that the cubic Li_2_MnTiO_4_ phase shows better structural reversibility and stability compared with the monoclinic one. Additionally, a small volume change was confirmed as one advantage of the cation-disordered material, which means there would be fewer mechanical problems such as deformation or fracture.
